# College students’ experiences, perceptions and management of health risks: a qualitative study in China

**DOI:** 10.3389/fpubh.2026.1765562

**Published:** 2026-03-20

**Authors:** Li Zhou, Weiqiang Qi, Zhujian Shao, Qing Zhou, Yu Zhang, Wenhao Yang, Lai Wei

**Affiliations:** 1 Department of Health Management, Zunyi Medical University, Zunyi, China; 2 Research Center for Humanistic Medicine, Zunyi Medical University, Zunyi, China; 3 Zunyi Medical and Pharmaceutical College, Zunyi, China; 4 Department of Health Economics, Zunyi Medical University, Zunyi, China

**Keywords:** college students, experiences, health risks, management, perceptions, qualitative study

## Abstract

**Introduction:**

The rising prevalence of non-communicable diseases (NCDs) among the youth in the world has raised the need for urgent intervention strategies. Despite this, young adults are often considered to be healthy by default and their specific health risks are thus understudied. This research has employed the qualitative approach in an attempt to explore the lived experiences, perceptions and strategies of college students in managing health risks.

**Methods:**

From March to April 2025, semi-structured and in-depth interviews were conducted with 39 students recruited from universities across China. The data collected were studied using thematic analysis by the help of NVivo software.

**Results:**

Thematic analysis identified four major themes: (i) Lived experiences of intersected risks of physical and psychological health; (ii) Cognitive appraisals of risk severity and personal susceptibility; (iii) willingness to change and underlying determinants; and (iv) Strategies utilized in health risk management, including systemic enablers and barriers. Participants described negotiating a complex landscape of health challenges, shaped by a dynamic interplay of personal, social and environmental factors.

**Conclusion:**

This study identifies the complex health risks experienced by college students and the complexities of how college students perceive and handle these risks. These challenges are not isolated but the result of interplay of individual behaviors, family influence, institutional policies and broader environmental contexts. The findings support a move towards comprehensive and socio-ecological approaches to the prevention of NCDs and the promotion of health in young adulthood.

## 1 Introduction

Non-communicable diseases (NCDs) are the leading cause of mortality and disability worldwide and the burden of NCDs is increasing among younger populations (1, 2). Data from the Global Burden of Disease Study 2019 highlight this trend by reporting that NCDs contributed to 38.8% of deaths in the 10–24 year old age group (3). A significant factor in this epidemic is modifiable behavioral risk factors, especially tobacco use, harmful alcohol consumption, physical inactivity and unhealthy dietary patterns (4). The consequences of these behaviors are dramatically demonstrated in terms of cancer outcomes: those with high risk behavioral profiles have a 29% greater incidence of cancer and a 52% greater mortality rate compared to those with low risk behavioral profiles (5). Accordingly, addressing these behaviors is at the heart of NCD prevention.

Because behavioral patterns that are established early in life set the stage for chronic morbidity and lower quality of life in adulthood (6, 7), addressing health-risk behaviors in youth has become an urgent global health priority. The university years, which are within this important window of early adulthood, are a particularly influential time for the formation and consolidation of health behaviors (8). Characterized by increased autonomy and less parental supervision, and exposure to new academic and social pressures, this transitional period can support the adoption of risky behaviors including unhealthy eating and substance use (9–12). At the same time, it is a good time to develop sustainable, health-promoting habits. Behaviors adopted during the university years may have long term consequences, with implications for long term health trajectories and the modulation of future NCD risk (13, 14). Thus, a sophisticated understanding of the determinants of behavior in this context is critical to designing effective interventions.

Despite a huge amount of epidemiological research documenting the prevalence of health risk behaviors among university students, there is still a huge gap in knowing the lived experiences, subjective perceptions, and practical ways to manage health risks among students. Quantitative studies, while useful in mapping prevalence and associations, are limited in their ability to offer insight into the complex, context-driven human factors, such as personal motivations, perceived barriers, and social influences, that ultimately influence behavioral choices. In order to go beyond what students do and understand why students do what they do, there is a need for qualitative exploration. This approach can help to explain how students perceive, interpret and negotiate health risks within their unique socio-ecological contexts.

To address this gap, the present study uses a qualitative design with an integrated theoretical framework to examine the health risks that college students face and how they perceive and manage their health risks in their socio-ecological environment. The overarching lens is the Theory of Triadic Influence (TTI), which conceptualizes behavior as arising from intrapersonal, social-contextual, and environmental streams (15). Within this structure, we include the Health Belief Model (HBM) to measure cognitive appraisals and the Risk Perception Model to measure affective dimensions of risk judgment. This integrated framework is used to guide all phases of the research process, from design to analysis.

## 2 Methods

### 2.1 Design

This qualitative study used in-depth, one-on-one, semi-structured interviews to investigate the experiences of college students, as well as perceptions and strategies of managing health risks. In order to ensure methodological rigor and transparency, the study was designed and reported in accordance with the Consolidated Criteria for Reporting Qualitative Research (COREQ) (16). The completed COREQ checklist is presented in Supplementary Appendix S1.

### 2.2 Ethics statement

Ethical approval for this study was obtained from the Medical Ethics Committee of Zunyi Medical University (Approval ID: [2025]3-003). The study was performed in strict accordance with the ethical principles of the Declaration of Helsinki (1964) and its amendments. All procedures involving human participants followed these and other relevant ethical standards and written informed consent was obtained from all participants before data collection.

### 2.3 Study sample and recruitment

A purposive sampling approach was used to recruit the participants who met the following inclusion criteria: (1) age 18 years or older, (2) currently enrolled in full-time undergraduate studies, (3) voluntarily agree to participate in the study, and (4) have the cognitive and communicative skills necessary for an interview. Exclusion criteria were (1) the diagnosis of a severe mental disorder that could hinder self-expression, and (2) withdrawal from the study before completion. In order to ensure a diverse range of perspectives, participants were chosen to provide variation in academic disciplines, institution type, gender, year of study and residential background. Of the 41 students approached, 39 were successfully enrolled—two declined participating, mostly due to scheduling conflicts or personal reservations.

### 2.4 Data collection

We used in-depth, one-on-one, semi-structured interviews to elicit rich and nuanced data about participant’s health risks. The development of the interview guide was done systematically taking into account both methodological rigor and depth. Its initial design was based on the integrated theoretical framework underpinning this study and comprehensive review of relevant literature, and then extensive discussions within the research team.

To enhance clarity and relevance, the preliminary interview guide was pilot tested with two college students that met the inclusion criteria but were not included in the final sample. Their feedback informed revisions to the wording, sequence and phrasing of questions. The guide was then finalized through consensus in the research team. The final version explored the following key domains:

1. What health risks do you think college students are in danger of?
2. Are you worried about your own health risks and what are the reasons? Do you underestimate your own health risks and if so why?
3. What health risks are hard for you to change, and what factors is it related to your decision to change?
4. What strategies do you have to cope with health risks? Have you engaged in health risk management and what are the factors that affect your implementation of these strategies?

Data was collected between March and April 2025. Interviews were conducted online by a team of seven research assistants, all students at the University with a stated interest in the topic. Their common academic background helped in building rapport and empathy with the participants. All assistants had prior experience in qualitative methods and were standardized trained by the research team to reduce interviewer bias and to ensure consistency.

Before each interview, participants were thoroughly briefed about the aims of the study, procedures and types of questions. They were assured of confidentiality and told that data would only be used for academic purposes. The concept of health risks was explicitly defined to include both personal factors (e.g., psychological, behavioral, lifestyle) and environmental factors (e.g., natural, interpersonal, social) to ensure a common understanding.

Following electronic informed consent, participants participated in private online interviews using video or audio call. Interviews were continued until data saturation was reached, the point at which no new thematic information was emerging (17). Each interview lasted about 30–40 min and was audio-recorded with permission of participants. Recordings were reviewed by the lead researcher (LZ), an experienced public health qualitative researcher and brief follow-up contacts were made if clarification of gaps or ambiguities was necessary. Participants were given an honorarium of 30RMB for their time.

Audio recordings were transcribed word for word within 48 h, and transcripts were given back to participants for member-checking to ensure accuracy. Data saturation was reached at the 39th interview, which was confirmed by team discussion after two more interviews that did not produce any new themes.

### 2.5 Data analysis

The thematic content analysis was used to analyze the data based on an integrated inductive and deductive reasoning. Although the coding process was open to the emergent themes of the data, the theoretical framework informed the interpretation of the findings.

NVivo software (Version 14) was used to help with data management. The coding was done by three researchers (LZ, WQ, and LW) of doctoral degree and with significant experience in qualitative research. The analysis process consisted of four significant steps:

1. Primary open coding: the first 5 interview transcripts were reviewed and inductively coded by two researchers (LZ and WQ) working independently. The emphasis of this stage was to base the analysis on the participant language in which the codes are created directly out of the data, and no pre-defined criteria are present.
2. Codebook development: the coders then checked, discussed and narrowed down preliminary codes. By agreement they came up with a structured codebook, where every code was well defined and explained with sample quotes of the transcripts to have uniform application.
3. Coding: systematic independent coding with the agreed codebook, the remaining transcripts were coded by all three researchers independently. The codebook was also dynamic in the sense that new codes could be added whenever necessary. Meetings were conducted on a regular basis to iron out the differences in coding through discussion until a point of agreement was achieved.
4. Theme generation and review: general themes were developed using synthesis of groupings of similar codes and examined through the theoretical framework. These themes were reviewed and narrowed down by the research team collectively to make sure that they were the most accurate representation of the data set and coherent to the research objectives.

### 2.6 Research rigor and trustworthiness

To make this qualitative study valid and credible, we followed the well-defined requirements of qualitative research and implemented the strategies in four major areas:

1. Credibility: To enhance the credibility and internal validity of our findings, we employed member checking. After the initial analysis, the participants were asked to peruse summaries and preliminary interpretations of their narratives so that our interpretation was based on their experience. Reflexivity by the researcher was also in practice; the team members frequently discussed and recorded their perceptions, backgrounds and potential impact on data collection and interpretation, mitigating personal biases by critically examining them and discussing them with colleagues.
2. Dependability: The structured audit trail and intercoder agreement guaranteed consistency and reliability. Transparent audit trail is allowed by the multi-phase analytic procedure (Section 2.5). The data were coded by three researchers working independently and frequent consensus meetings were conducted to decide on the coding discrepancies and high levels of interpretive consistency and analytical stability were reached.
3. Confirmability: To ensure that research was done on data, rather than on bias of the researcher, there was the use of multiple coders and audit trail. Any interpretation was strictly connected to raw data using direct quotes of the participants that are presented in the results. The other aspect that supports confirmability is the recording of the decision-making process during the process of analysis.
4. Transferability: Adequate information on the context was also given to enable the reader to determine whether the findings can be applicable to other situations. This entailed detailed descriptions of the characteristics of the participants, the context of the study, the data collection methods and the theoretical framework used to ensure that other readers can determine whether the results can be applied to other situations or not.

## 3 Results

### 3.1 Participant characteristics

The overall number of people in the study was 39. Table 1 summarizes their demographic characteristics. The sample consisted of 46% males and 54% females students. Regarding grade level, freshmen had the largest group (38%), followed by juniors (36%) and sophomores (21%). In terms of geographic background, 62% participants were from rural areas, while 38% were from urban areas.

**Table 1:** Demographic characteristics of participants (*n* = 39).

Variable	Number (%)
Gender	
Female	21 (54%)
Male	18 (46%)
University year	
Freshman	15 (38%)
Sophomore	8 (21%)
Junior	14 (36%)
Senior	2 (5%)
Major	
Humanities	18 (46%)
Science	5 (13%)
Engineering	8 (21%)
Medicine	8 (20%)
School	
Key school	11 (28%)
Non-key school	28 (72%)
Residence	
Urban	15 (38%)
Rural	24 (62%)

### 3.2 Themes

Four overarching themes were identified in the data analysis, which reflect the core dimensions of students’ engagement with health risks: (i) lived experiences of intersecting physical and psychological health risks; (ii) cognitive appraisals of risk severity and personal susceptibility; (iii) willingness to change and underlying determinants; and (iv) health risk management strategies, and systemic enablers and barriers. Each theme is elaborated in the following sections, with direct quotes from participants (identified by codes—e.g. P1).

#### 3.2.1 Theme 1: lived experiences of intersecting physical and psychological health risks

This theme reflects the various health risks faced by college students such as unhealthy lifestyle habits, psychological stress, addictive behaviors, and environmental hazards, and the multidimensional nature of health challenges that are pervasive in the daily lives of college students. These cumulative exposures are important, yet modifiable, determinants of non-communicable and communicable diseases.

##### 3.2.1.1 Sub-theme: unhealthy lifestyle habits

Among unhealthy lifestyle habits, the most common risks reported were staying up late (*n* = 31), unhealthy diets (*n* = 21) and lack of exercise (*n* = 17). These behaviors often came about because of academic pressures, social norms, and the need for convenience, and are a mixture of intrapersonal and institutional factors.

- Staying Up Late: Many students said they were sacrificing their sleep for short-term pleasure, such as watching TV series or playing games late at night. As one participant explained, *“Most college students nowadays are fond of staying up late to watch TV series and play games.”* (P3).
- Unhealthy Diet: Dependence on takeout food, due to its convenience and affordability, was another common risk, especially among students with busy schedules. One participant pointed out, *“Many students have takeout food for convenience … Most of these takeout meals are high in oil and salt*.*”* (P39).

##### 3.2.1.2 Sub-theme: psychological health risks

Psychological health risks were also reported widely, with some of the major sources of stress being employment concerns (*n* = 14), social barriers (*n* = 14), emotional distress (*n* = 10), academic pressure (*n* = 5) and relationship difficulties (*n* = 3). Students’ emotional fluctuations were often related to both immediate challenges, such as academic workload, as well as longer term worries about the future.

Social and Emotional Stress-Many students struggled to balance social and emotional demands and academic responsibilities. One participant explained, *“College students may experience emotional fluctuations including problems related to their studies and personal emotional issues. Some students also have a problem with socializing.”* (P35).

##### 3.2.1.3 Sub-theme: addictive behaviors

Addictive behaviors were common among students, including smoking (*n* = 11), alcohol consumption (*n* = 11) and unsafe sexual behavior (*n* = 2). These behaviors were highly influenced by social norms, peer pressure, and decreased parental supervision that is typical of the college setting.

- Smoking and drinking: smoking and drinking were seen as normal activities and often encouraged by easy availability of substances and peer influence. One participant described, *“Every day after class I can see many people smoking in the smoking area* … *Smoking, drinking alcohol and an unhealthy diet are the things that I most frequently come across around me.”* (P33).
- Unsafe sexual behavior: unsafe sexual practices which increased the risk of sexually transmitted infections including HIV were also reported. The normalization of casual sex, as well as a lack of education about safe practices contributed to this health risk.

##### 3.2.1.4 Sub-theme: environmental health issues

Environmental risks such as air pollution and secondhand smoke were also identified, but less commonly (*n* = 4). College students are especially at risk for secondhand smoke, which is still prevalent in many university settings, especially in areas where smoking is prevalent.

- Air pollution: in cities with high levels of air pollution, students expressed concern for long-term health effects of exposure. One participant commented, *“Haze is a big problem in the city I live in and everyone is very worried about its effect on health.”* (P32).
- Secondhand smoke: some students reported consistent exposure to secondhand smoke in public spaces or designated smoking areas on campus. As one participant stated, *“Many college students are still subjected to secondhand smoke.”* (P7).

#### 3.2.2 Theme 2: cognitive appraisals of risk severity and personal susceptibility

##### 3.2.2.1 Sub-theme: concerns and reasons regarding health risks

Some students (*n* = 11) indicated that they had no concerns about their health risks. They suggested advanced medical technology and their own proactive behaviors made these risks seem more manageable: *“Since the current medical technology is quite advanced, and I also engage in regular exercise, I do not worry too much about health risks.”* (P26). Others felt protected as they were asymptomatic and *“still young with a strong recovery ability”* (P36) which made them less urgent to change.

On the one hand, a substantial number of the participants (*n* = 28) reported clear concerns about their health. These concerns were often triggered by personal experiences of illness, exposure to media reports of sudden death or serious disease, or observation of health problems in family members and peers. For example, one student said, “*When I read the news about people dying suddenly due to staying up late. I will be worrying if I might die suddenly.”* (P27). Others directly linked health to their life goals and future plans: *“Only when I am in good health I can do the things I want to do.”* (P39). A strong sense of moral and emotional responsibility towards family was also evident with some students expressing worry of the financial and emotional burdens that their illness might impose on their parents: *“Getting ill makes family members worry and increases family costs.”* (P14). Several participants highlighted the uncertainty in health risks and expressed fear or anxiety related to situations that are out of their control: *“Health risks often come with uncertainty … and I may experience fear and anxiety about the out-of-control situation.”* (P34).

##### 3.2.2.2 Sub-theme: underestimate and reasons regarding health risks

Some of the students (*n* = 19) underestimated their health risks. Reasons for this included a belief that youth provides protection (“youth can withstand anything”), optimism bias (“bad things will not happen to me”), limited knowledge of risk factors and a tendency to focus on immediate enjoyment rather than long-term health. Typical comments included: *“I thought bad things would not happen to me.”* (P32), *“As long as I do not stay up continuously for several days, it will be okay.”* (P2) and *“youth can withstand anything.”* (P8). Some explicitly framed indulgence and hedonism as inherent to being young: *“Because I am still young, indulging in pleasures is a characteristic of young people.”* (P36). Others recognized that a lack of knowledge made it hard to fully understand the harms of their behaviors: *“I have insufficient knowledge about health risk factors … It would be hard for me to realize the harm of my own behaviors to my health.”* (P34).

At the same time, some students (*n* = 20) said that they did not underestimate their health risks. They attributed this awareness to having strong personal health values, having more health knowledge, and direct or vicarious experiences with illness. One participant summarized well, *“I attach great importance to health issues.”* (P21), while another linked risk awareness to a fear of mortality: *“Because I’m rather afraid of death.”* (P31). For some, the study of medicine or health-related subjects fundamentally changed the way they think: *“Before I studied medicine, I would underestimate my own health risks … However, once I began the study of medicine. I have started to increase my awareness of health risks.”* (P33). Having or seeing health issues caused by unhealthy lifestyles also contributed to a more cautious approach: *“I have also seen many cases where health problems occurred due to poor living habits.”* (P6).

#### 3.2.3 Theme 3: the willingness to change and its underlying determinants regarding health risks

##### 3.2.3.1 Sub-theme: hard-to-change health risks and the willingness to change

Participants admitted that some health risks were especially hard to change, such as staying up late (*n* = 27), unhealthy eating (*n* = 5), physical inactivity (*n* = 5) and alcohol consumption (*n* = 3). In the face of these types of challenging behaviors, a small number of students (*n* = 6) openly articulated that they had no immediate intention to change, whereas the majority (*n* = 33) described an ongoing struggle between the desire to improve and the difficulty of taking consistent action.

##### 3.2.3.2 Sub-theme: obstacles that prevent students from wanting to change

At the individual level, students identified several barriers to changing their health risks. These included biased perceptions of risk, a tendency to focus on short-term pleasures, and long-lasting habits and a lack of self-discipline. Many reported unhealthy routines brought immediate psychological or physical comfort: for instance, unhealthy diets were associated with tastiness, and being up late to watch dramas or playing games was perceived as a way of relaxing and rewarding themselves. As one student explained, *“I do not feel that staying up late has brought about any noticeable physical changes in me yet … and staying up late sometimes makes me happy, so I will not change it for now.”* (P2). Others pointed to entrenched habits and personal traits as major obstacles: *“Lack of self-discipline and a tendency to take risks. … failed to make any changes.”* (P27). Some students referred to laziness, sitting for long periods, and excessive use of electronic devices as part of long-established routines that were not easy to break: *“My own laziness … hindered me from making changes.”* (P3); *“Sitting for long periods and excessive use of electronic devices have become my long-established habits.”* (P39).

Environmental temptations and structural constraints also hindered students’ willingness to change. Some highlighted regional dietary cultures and the local food environment as a barrier to improving their eating habits: *“I’m in Xinjiang … the dietary habits are also different … so it’s quite difficult for me to make a change.”* (P24). Others commented about the availability of cheap, high-fat and high-salt foods on campus, especially when having meals with classmates: *“There are many dining options near my school, and I often have some dishes with high oil and salt content when having meals with my classmates.”* (P36). Academic pressure was another common hurdle; overloaded schedules, workloads and emotionally taxing study habits left little room for sleep, exercise or meal planning. As one participant explained, *“Due to the heavy workload and academic demands at present I have to reduce my sleep time to achieve better grades.”* (P38).

##### 3.2.3.3 Sub-theme: factors that prompt someone to want to change

Alongside these barriers, students identified factors that helped or motivated attempts to change. Individual-level facilitators included aspirations for a healthier life, recognition that current habits were impacting daily functioning, and health knowledge and awareness. Some students said that recognizing the negative effects of unhealthy behaviors led to them setting goals to improve: *“Bad habits have indeed had an impact on my life; I have expectations for a healthy lifestyle.”* (P20). For others, studying health-related majors helped keep up motivation: *“As a medical student, I want to improve my overall condition. I also want to decrease my health risks.”* (P33).

Interpersonal influences were also found to facilitate health risk management. Feedback and reminders from their peers or family members sometimes increased student awareness of risks and behavioral change. For example, one participant reflected on being told that their weight was slightly above a healthy range and this prompted consideration: *“… others have told me that my current weight is a bit too high and I should lose some weight.”* (P25). Others showed concern that habits like staying late or making noise in the dormitory might have a negative impact on those around them, which motivated them to adjust their routines: *“I’m afraid staying up late might have an impact on the people around me.”* (P19). At a more general social level, information about health risks in the media or seeing the health problems of others were powerful reminders. One student explained his or her decision to change after *“seeing the social news about diseases and experiencing the adverse symptoms caused by staying up late.”* (P15).

#### 3.2.4 Theme 4: deployed risk management strategies and enablers or barriers

##### 3.2.4.1 Sub-theme: strategies for managing health risks

For physical health risks, students used strategies such as time management, self-discipline, and social support. Many reported setting clear goals and planning their routines in order to promote healthier behaviors. For example, some devised daily schedules to control sleep and physical activity: *“I will make a daily time management table for myself. I will go to sleep before 24:00 …”* (P27). Others used technology to track their health, for example, tracking their weight: *“I will monitor my weight management by downloading software.”* (P28). To reinforce self-discipline, some students had set specific goals to work towards in relation to their health, and sought to be accountable through social support: *“I will strictly formulate a plan and find a partner to supervise together.”* (P33).

In dealing with mental health risks, students used both emotion-focused and problem-focused coping strategies. Many focused on regulating their emotions, practicing self-awareness, or getting emotional support from others. Some reported having developed specific emotional regulation skills: *“I have practiced emotional management and learned to recognize my own emotions.”* (P12). Others relied on strategies like entertainment or socializing to relieve stress: *“When I find myself in psychological pressure. I will choose to temporarily escape by watching a favorite American TV series to relax.”* (P37). Social support also turned out to be an important coping mechanism: *“When I’m not in a good mood, I usually call someone and have a video chat with them.”* (P33). Additionally, several students described actively growing their social networks as a way to cope with mental health challenges: *“I will make myself brave enough to socialize, expand my social circle.”* (P3).

##### 3.2.4.2 Sub-theme: obstacles and facilitators of health risk management

While students used a variety of strategies to deal with their health risks, they also encountered various barriers and enabling factors. Although most participants (*n* = 30) reported that they actively engaged in health risk management, a subset of students (*n* = 9) did not. These obstacles and facilitators are described in the following thematic section.

Key facilitators of health risk management were health knowledge, societal promotion of healthy lifestyles and individual aspirations for long-term well-being. Several students highlighted the importance of gaining knowledge, either through formal education or online health content, in terms of triggering healthier behaviors: *“I also took the course on Chinese culinary culture and learned how to maintain a balanced diet.”* (P1). Societal emphasis on healthy living, often promoted by media figures, further reinforced positive behaviors: *“There is a growing emphasis on a healthy lifestyle in society, and some video bloggers have created videos on the topic.”* (P17). Additionally, the aim to have a better future and self-improvement was also a great motivator: *“I need to maintain good health so that I can face tomorrow and become the best version of myself.”* (P26).

However, there were both internal and external barriers that hindered consistent and effective health risk management. Internal barriers included low levels of health knowledge, cognitive biases and the preference for short-term pleasures over long-term well-being. Many students said they experienced a conflict between the desire to have immediate rewards and the desire to choose healthier options in the future. For example, some considered health risk behaviors to be sources of instant gratification or to be ways of dealing with stress: *“I prioritize short-term pleasures, such as staying up late to watch TV series or eating takeout food.”* (P8). Others described discomfort and demotivation when trying to practice healthy habits, such as dieting or exercising: *“Sometimes I feel that exercising and controlling my diet are self-torture. When I’m keeping my diet going, I’m in a pretty low mood.”* (P32). In addition, some students viewed the risks of unhealthy behaviors as not being immediately severe or as not being severe at all, believing that their youth offered protection: *“The risks to my physical and mental health were not severe.”* (P10); *“Perhaps it’s because I still feel young and believe that the physical burden is within my capacity.”* (P16). A lack of self-control and practical knowledge about managing risks was also significant barriers: *“I do not know how to manage the risk factors related to mental health.”* (P24); *“I lack self-control and often have no motivation to keep going.”*(P23).

External barriers, such as academic demands and environmental factors, limited students’ capacity to manage their health risks. High academic workloads and lack of time for relaxation, exercise or socializing were common reasons: *“Our study load is heavy, and we have little time for entertainment, relaxation, and exercise”* (P13). Environmental temptations, such as the widespread availability of unhealthy food on campus and peer influences, further hampered the efforts of students: *“There are lots of delicious foods around my school, and it’s very convenient. I am also influenced by my roommate’s unhealthy lifestyle.”* (P36).

## 4 Discussion

Based on the thematic analysis, this qualitative research involved the investigation of the lived experience, perceptions, and health risk management of college students. Instead of assuming that risk behaviors exist as isolated phenomena, we have found four themes that are interconnected and contribute to the description of a complex and multi-level health risk environment: (i) multifaceted health risks embedded in academic and campus life; (ii) ambivalent risk perceptions that oscillate between concern and normalization; (iii) multilevel barriers and facilitators of the willingness of students to change health risks; and (iv) pragmatic but fragmented health risk-management behaviors. Through these themes, we were able to show how personal cognitions and feelings, interpersonal relationships, and institutional and environmental factors interact to influence the health-related decision-making process of students and their ability to cope with risks.

By providing a holistic view of lived risks, our results are a contribution to prior studies on the health risks of college students that have largely concentrated on individual behaviors or have been quantitative in nature. We also shed light on how these risks are perceived and bargained by students, and why behavior change has been difficult even in situations where risks have been clearly identified. It is worth noting that the paradox of concern to normalization among students in their risk perception along with the conflict of long-term health expectations and short-term academic and social expectations supports the necessity of multi-level and context-specific interventions. These efforts should not be focused on individual health literacy and motivation but on the socio-institutional structure of university life in general.

The most common health risks that were reported in the present study included maladaptive lifestyle behaviors especially voluntary sleep deprivation, unhealthy diets, lack of exercise, and excessive sedentary lifestyles. Adequate sleep is the basis of physical and mental health; however, a high percentage of students regularly trade sleep to recreation or to manage the high academic loads (18). Notably, interviewees explained staying up late not only as a self-control issue but as a way of catching up with the academic work or relaxing after a hard course work. This implies that sleep deprivation is thoroughly rooted in larger trends of academic pressures and coping. In turn, the lack of systemic changes to the curriculum design, the intensity of assessment, and the current culture of overwork on campus can make interventions that promote sleep hygiene ineffective.

Likewise, the mentioning of poor diets and a lack of physical activity is in line with the literature that shows that the quality of diet in college students tends to be of lower quality than in older adults due to a complex interaction of personal, family, institutional, and social factors (19, 20). Students emphasized the convenience and social usefulness of takeaway food, the availability of low-cost, energy-dense foods in the college food landscape. The lack of exercise was frequently viewed as an inevitable factor because of the requirements of studying on the screens, which were aggravated by tiredness and lack of motivation to exercise (21). These stories emphasize the significance of interventions at the environmental level. Colleges should cease to focus on health education that is targeted at each student and instead take a proactive role in transforming campuses, such as subsidizing available healthy and nutritious foods and making physical exercise a regular part of academic life.

Substance abuse and especially smoking and alcohol consumption were common and deeply rooted in social practices, which compounded threats to the health of students greatly. As it has been reported previously, smoking and drinking were often co-morbid and overlapping in personal and social determinants (22, 23) and thus these two behaviors can be better described as components of a larger behavioral syndrome than as discrete habits. The majority of our sample students indicated that they participated in two or three simultaneous risk behaviors, which is consistent with quantitative research in the clustering of unhealthy lifestyles in young adults (24, 25). Since the interactive implications of various maladaptive behaviors tend to be greater than the aggregate effects of each (26), the treatment of comorbid risks is a vital public health concern.

In theory, such qualitative observations are highly reminiscent of the Problem Behavior Theory, which assumes that the development of youth involvement in risk behavior is the result of the interaction between individual factors, immediate social conditions, and the acquisition of behavioral patterns (27). Moreover, the results of our research coincide with the multi-level approach to the TTI. The narratives of students clearly outlined how individual interests, peer influence, campus climate, and the larger social communication combine to influence substance use and other risk behaviors. Going beyond the paradigms of individual blame, our findings suggest coordinated, ecological interventions that would be working at the individual, familial, peer, and community levels to break the clustering and normalization of such behaviors.

In terms of psychological health, previous studies prove that depression is common in college groups, and the prevalence among Chinese college students is much higher than in the general population (28, 29). We have found that students are exposed to various overlapping stressors regarding academic achievements, daily logistics, interpersonal relationships, and employability in the future. Accumulation of stress over time may lead to depression, anxiety and learned helplessness. In this population, psychological distress does not only represent a specific mental health issue; it also serves as a predisposing factor to other health-related hazards, making them more susceptible to infectious illnesses, substance abuse, and suicide attempts (30). The psychological risks which were identified in this study have a deep structural basis especially employment instability and social barriers. Mental health support of this population needs to be holistic and take advantage of the available internal and external resources (31). The interventions must not only be on the reduction of the symptoms but also on the upstream determinants of mental health such as vocational support and social isolation reduction initiatives. The implications of these findings is that development of intrinsically supportive university environments is very important. Career counseling, social skills training and ready access to mental health services should be integrated into the mainstream campus life, not as marginal services, to assist students in coping with the transition into adulthood.

Second-hand smoke (SHS) exposure became an important issue in environmental health, especially in and around the dormitories where smoke-free policies were seen as not being strictly implemented as in other social places. Although it is important to reduce the overall air pollution by implementing macro-level efforts in society, strictly implementing smoke-free policies in college dormitories, along with specific education about the dangers of SHS, should be seen as a viable measure towards minimizing the environmental health risks on campus.

Majority of the participants were concerned about the health risks especially about the immediate effects of sickness and the overall effects on their future planning and families. Students were also concerned that ill health would curtail their potential in life or subject their parents to emotional and financial stress. These expected results were usually used as a major point of reference during risk assessment. This is in line with studies that have found that people estimate risks in relation to their knowledge of the possible negative outcomes, and that these estimates can induce defense (32, 33). Appeals to responsibility on behalf of one self and on behalf of family well-being were found to enhance the sense of health responsibility in students of our study and in some cases triggered proactive risk-management practices.

Ironically, these risks were underestimated by a number of students at the same time, and they used their physical vitality and youth as the signs of the inherent resilience. Dependence on the perceived safety net of advanced medical technology further reduced the focus on preventive care. Almost 50% of students directly underestimated their individual vulnerability, which is also in line with the well-established phenomenon of the optimistic bias, whereby people view their risks as less than those of other people (34, 35). In case of risk underestimation, people might be less inclined to take precautionary steps, which can jeopardize the health results in the long run (36). We have found that subjective factors which included optimistic bias, focus on immediate enjoyment, and perceived personal irrelevance affected underestimation more than objective information. The health promotion strategies should therefore shift away to fear-based or simply informational messages, but they should use positively framed, future-oriented messages that directly relate the daily habits to long-term personal and family objectives of the students.

In terms of readiness to alter behavior, the students reported that there was a strong intention-behavior gap: despite the recognition of risks, breaking the established habits demanded overcoming physiological and psychological addictions, including the sacrifice of the comfort and social capital that these habits gave them. Since university life is unique in terms of stressors and increased sensitivity to peers, institutional support is necessary. Long-term self-regulation can be achieved through developmentally appropriate health education, communication with the family, and easy access to interventions. Students underlined the importance of environmental temptations (e.g., the ubiquity of unhealthy food, normative social drinking) and structural limitations (e.g., academic overload, the lack of exercise facilities) in their day-to-day decisions. Therefore, framing behavior change as a challenge of personal will is insufficient, and universities should develop choice environments that would ensure that the healthiest options are the most visible, acceptable, and feasible.

Students used both internal and external measures in regard to the management of physical health risks. Self-monitoring (e.g., scheduling, health-tracking apps) and goal-setting were often used, and peer accountability was often added to them. Since peer dynamics are a significant force during the young adulthood, peer-based interventions are a promising and culturally acceptable method of setting up healthier campus norms.

Emotional-focused (e.g., emotional regulation, leisure-based escapism) and problem-focused (e.g., seeking professional or social support) strategies were applied to manage mental health risks by the participants. According to previous studies, coping styles have a considerable impact on mental health outcomes (37). Emotional and problem-oriented approaches can be used in a complementary fashion: the former are important in dealing with short-term distress, whereas the latter are essential when stress surpasses the internal coping ability. Moreover, the external support is not only helpful in solving the problem in practice but also leads to a feeling of connectedness and belonging to the institution (38).

The majority of the students have indicated that they were involved in some type of health risk management, which was highly influenced by internal and external forces. In line with past studies, risk perception was found to be a major predictor of health-related action (32, 33). We have found that less perceived risk was linked to more underestimation, less expressed concerns, and less motivation to change. Thus, the development of risk perception interventions is necessary. The pattern of concern-normalization that was found in the narrations of the students, however, indicates that the increase in risk perception might not be enough: increased concern can co-exist with risky behavior, especially when the behavior is a way to relieve or feel pleasure or a sense of normalcy. Most respondents explained that some health risks provided physical or psychological relief or had become a source of dependency and behavioral change was not easily attained without further support.

Lack of mental health literacy became a significant impediment to the management of psychological risks. Some of the students did not know how to recognize mental health issues or how to use the institutional support system. Since the concept of mental health literacy involves not only the ability to recognize symptoms, but also coping strategies and treatment options (39, 40), our findings support the importance of comprehensive educational strategies. The systematic implementation of the process of seeking help and equipping students with early signs of distress should be a part of the academic curriculum.

This research has a number of weaknesses. To begin with, voluntary recruiting is inherently self-selected, so the students who already have health awareness or have strong views on campus health can be over-represented, which can leave out the views of highly disengaged students. Second, due to the nature of the current study, which took place in the socio-cultural and learning environment of Chinese universities, it is possible that such results cannot be generalized to other higher education systems that have different cultural norms. Third, qualitative interview data is prone to recall bias and social desirability. Although there was an assurance of confidentiality and an attempt to promote candour, there is the possibility that the participants retrospectively rationalized or positively framed their behaviors. Lastly, as per the nature of thematic analysis, these results are an interpretive narrative that is influenced by the theoretical view of the researchers, even though there were strict mitigation measures that were implemented such as multiple coding and consensus building. Despite these shortcomings, this work offers detailed, contextual information on the health risks of college students as a strong theoretical basis of future longitudinal and mixed-method studies.

## 5 Conclusion

This research paper will show that students are exposed to a compounding and overlapping web of physical and mental hazards that are deeply rooted in the academic setting. Notably, our results indicate that there is a paradoxical nature of student cognition: though the majority of the respondents showed their real concern, declared their willingness to change their behavior and tried different risk-management methods, they also exhibited a widespread optimistic bias, which invariably underestimated their personal vulnerability and the seriousness of their habits. It is not merely a matter of willpower that students can translate intentions into sustained action with regard to health but rather a very deep socio-ecological interaction of the internal (e.g., health beliefs, motivation, self-efficacy, and emotional regulation) and external (e.g., family dynamics, peer norms, institutional policies, and the wider socio-physical environment) factors. In turn it is not sufficient to conceptualize health risk management as an individual responsibility. Higher education institutions must be holistic and multi-level in order to effectively protect and support the well-being of students. These interventions need to go beyond the single behavioral education to actively change the campus setting, include health promoting institutional policies, and to mobilize the family and peer support systems.

## Data Availability

The datasets presented in this study can be found in online repositories. The names of the repository/repositories and accession number(s) can be found at: DOI: 10.6084/m9.figshare.30530033.
